# The Scoliosis Research Society adult spinal deformity standard outcome set

**DOI:** 10.1007/s43390-021-00334-2

**Published:** 2021-04-06

**Authors:** Marinus de Kleuver, Sayf S. A. Faraj, Tsjitske M. Haanstra, Anna K. Wright, David W. Polly, Miranda. L. van Hooff, Steven D. Glassman, the COSSCO study group, Ahmet Alanay, Saumyajit Basu, Shay Bess, Darrel Brodke, Leah Y. Carreon, Marinus De Kleuver, Helton L. A. Defino, Sayf S. A. Faraj, Steven D. Glassman, Martin Gehrchen, Munish C. Gupta, Tsjitske M. Haanstra, Yong Hai, Henry F. M. Halm, Ian Harding, Virginie Lafage, Gabriel Liu, Morio Matsumoto, Ibrahim Obeid, Stefan Parent, Ferran Pellisé, Howard M. Place, David W. Polly, Dominique A. Rothenfluh, Rajiv Sethi, Maarten Spruit, Lewis J. Stephen, Juan S. Uribe, Miranda L. Van Hooff, Anna K. Wright, Mitsuru Yagi, Zezhang Zhu

**Affiliations:** 1 Department of Orthopedics, Radboud University Medical Center, Postbus 9101, 6500 HB Nijmegen, The Netherlands; 2 Research, Sint Maartenskliniek, Nijmegen, The Netherlands; 3 Neuroscience Institute, Virginia Mason Medical Center, Seattle, WA USA; 4 Present Address: Fred Hutchinson Cancer Research Center, Seattle, WA USA; 5 Department of Orthopaedic Surgery, University of Minnesota, Minneapolis, USA; 6 Department of Orthopedic Surgery, Norton Leatherman Spine Center, University of Louisville, Louisville, KY USA; 7 Association of Dutch Burns Centers, Beverwijk, The Netherlands

**Keywords:** *Outcomes assessment*, Core outcome set, *Spine*, *Delphi technique*, *Patient-reported outcomes*, Adult spinal deformity

## Abstract

**Purpose:**

Symptomatic adult spinal deformity (ASD) with an extremely variable presentation with pain, with and without neurogenic leg pain, and/or disturbed sagittal and coronal balance, causes a significant societal burden of disease. It is an important consequence of the aging adult population, generating a plethora of spine-related interventions with variable treatment efficacy and consistently high costs. Recent years have witnessed more than a threefold increase in the prevalence and treatment of ASD, and further increases over the coming decades are expected with the growing elderly population worldwide. The ability to monitor and assess clinical outcomes has not kept pace with these developments. This paper addresses the pressing need to provide a set of common outcome metrics for this growing group of patients with back pain and other disabilities due to an adult spinal deformity.

**Methods:**

The standard outcome set was created by a panel with global representation, using a thorough modified Delphi procedure. The three-tiered outcome hierarchy (Porter) was used as a framework to capture full cycle of care. The standardized language of the International Classification of Functioning, Disability and Health (WHO-ICF) was used.

**Results:**

Consensus was reached on a core set of 25 WHO-ICF outcome domains (‘What to measure’); on the accompanying globally available clinician and patient reported measurement instruments and definitions (‘How to measure’), and on the timing of the measurements (‘When to measure’). The current work has brought to light domains not routinely reported in the spinal literature (such as pulmonary function, return to work, social participation), and domains for which no adequate instruments have yet been identified (such as how to clinically quantify in routine practice lumbar spinal stenosis, neurogenic claudication, radicular pain, and loss of lower extremity motor function).

**Conclusion:**

A standard outcome set was developed for patients undergoing treatment for adult spinal deformity using globally available outcome metrics. The current framework can be considered a reference for further work, and may provide a starting point for routine methodical and systematic monitoring of outcomes. Post-COVID e-health may accelerate the routine capture of these types of data.

**Supplementary Information:**

The online version contains supplementary material available at 10.1007/s43390-021-00334-2.

## Introduction

Symptomatic adult spinal deformity (ASD) is an important consequence of the aging population, generating a plethora of spine-related interventions with variable treatment efficacy and consistently high cost. While recent years have witnessed more than a threefold increase in the prevalence and treatment of ASD [1], further increases over the coming decades are expected globally [2, 3]. Increasing health and economic burden has led to policymakers and payers putting more emphasis on value driven healthcare, which includes the patient’s perspective [4].

Collectively described as ‘adult spinal deformity’, ASD has heterogeneous presentation, and multiple etiologies, encompassing spinal deformities originating in childhood (e.g., congenital, idiopathic scoliosis) and those developing later in adulthood, such as post-trauma [3]. However, the large increase in prevalence of ASD is mostly due to late-onset deformities in the often frail aging population [3]. Progressive deformities of the spine and trunk result in a loss of body height, a stooped posture which we can observe around us in everyday life, and spinal stenosis [5]. Symptoms that commonly occur are back pain, neurogenic leg pain, and fatigue due to the increased energy expenditure required to maintain an upright posture and gait. The impact on Health-Related Quality of Life (HR-QoL) and psychological wellbeing, often underestimated, is demonstrably worse than other chronic conditions such as congestive heart failure and chronic lung disease [3, 6].

To improve the quality of life or to prevent further deterioration, both noninvasive and invasive interventions are utilized. These may include self-management, physical therapy, injections, spinal canal decompression, and instrumented corrective fusions. Although evidence exists that some of these interventions can be effective in selected patients [7, 8], and appropriate use criteria (AUC) have been identified [9, 10], which treatment will benefit which patient the most is still poorly defined. This is due to the heterogeneity of the often frail ASD population, complex decision-making, and the lack of consistent standardized outcome measurement. As a result, when comparing findings from different studies and registries, it is not possible to draw conclusions regarding the effectiveness of its interventions.

A feedback loop involving routine measurement of outcomes across the full cycle of care is a prerequisite for improvement of quality of care and for evidence-based decision-making concerning cost effectiveness and innovation [4]. Outcome measures should, therefore, be standardized to be comparable, risk-adjusted, reporting on short- and long-term health outcomes, and should include measures of disability, ability to work, and social participation [11, 12]. Once identified, this data set can be implemented in (Electronic) Medical Records. This allows for visualization for patients and caregiver, for individual shared decision making and direct patient feedback at follow-up. Data can also be aggregated for the institutional Plan-Do-Study-Act (PDSA) quality improvement cycle. And ultimately, because it is standardized, the data are mergeable with other institutional and registry data. The aim of this study was to take the first step, and establish such a universal standard outcome set, using internationally recognized frameworks and languages, and employing as many existing and recognized measurement instruments. This standard outcome set combines patient-reported (e.g., pain) and clinician-reported outcomes (e.g., 30-day readmission) for all ASD patients undergoing a surgical intervention.

## Materials and methods

### Scope

This project was registered in the Core Outcome Measures in Effectiveness Trials (COMET) database (ID 1343) [13], and guidelines for the development of a core set of outcomes were applied [14]. These well recognized guidelines require a procedure in which a preparatory systematic review is followed by a formal consensus procedure (modified Delphi). A formal consensus study is an appropriate methodological design, based on both qualitative and quantitative methods, which makes it is possible to bridge the gap in knowledge from the literature, (i.e. which outcome domains and measures should be used to compare study results and evaluate the quality of care) with internal knowledge (based on experience of experts).

The Core Outcome Set standards for reporting (COS-STAR Statement) were used to report the study findings [15].

The three-tiered outcome hierarchy was used as a framework to capture full cycle of care [16]. This is endorsed by the International Consortium for Health Outcomes Measurement (ICHOM) [17] and the Organization for Economic Cooperation and Development (OECD) Health Ministers [18]. The standardized language of the International Classification of Functioning, Disability and Health (WHO-ICF) was also used [19].

### Project team

A project team consisting of methodologists and spine surgeons/specialists, not participating in the Delphi rounds, was assembled to conduct the study.

### Panelists

Twenty-five experts from the Scoliosis Research Society (SRS; clinicians and researchers) with at least 5 years’ experience in the treatment or research of spine deformity were included as panelists [North America (*n* = 10), Latin America (*n* = 1), Europe (*n* = 8) and Asia–Pacific (*n* = 6) (Supplementary Material Fig. 1)].

### Delphi procedure (Fig. 1)

**Fig. 1:**
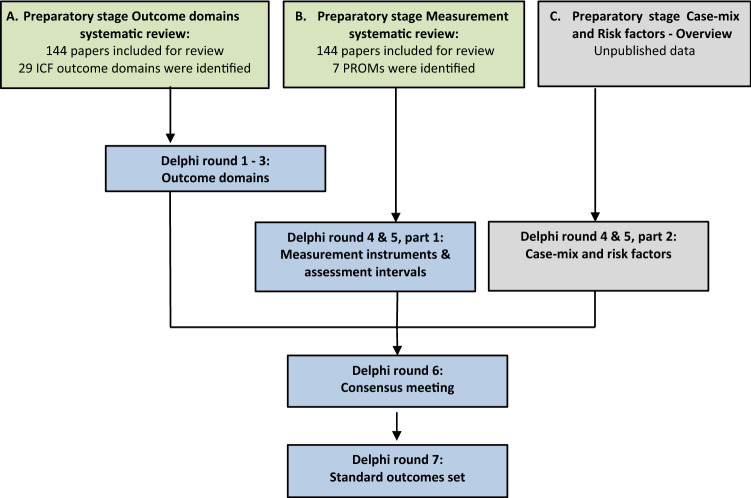
Workflow of the modified Delphi process. Blue boxes refer to work of current paper. Green boxes refer to a previously published systematic review [20]. Grey boxes are described in methods section under “Delphi procedure”, but due to complexity and the required descriptions the relevant case-mix and risk factors are out of scope of this study

A systematic literature review of 144 papers was performed to identify current patient-reported outcome domains and corresponding patient-reported outcome measures (PROMs) in ASD surgery [20]. These 29 domains were classified according to the WHO-ICF [19] and served as input for the first Delphi round.

For round 3, an overview of 37 clinician-reported outcomes (e.g. nerve root injury, surgical site infection, 30-day re-admission) was made available to all the panelists. These outcomes were derived from multiple published sources, including single-center and multi-center studies, validated risk stratification tools, comprehensive systematic reviews, studies from the American College of Surgeons’ National Surgical Quality Improvement Program, and the LBP set of ICHOM [17].

The panelists were asked to answer “Yes or No” to questions related to outcome domains and measurement instruments (patient-reported and clinician-reported) and they were asked to consider their own professional opinion as well as evidence provided from the literature. They were encouraged to provide free text feedback.

After each round, an anonymized feedback report was generated and made available to the panelists, with an overview of votes and panelist’s feedback, comments, and adaptations made to the list of potential items. This report was used as input for subsequent rounds. The threshold for consensus was set at ≥ 75% agreement. In accordance with COS guidelines [14], items with 50–75% consensus were made available again for voting in the subsequent round, and additional information from the literature was provided to make an informed decision. Items with < 50% agreement and/or repeated lack of consensus were excluded.

After reaching a consensus on ‘what’ to measure (Delphi round 1–3; response rates 25/25, 25/25, 24/25, respectively), the panelists voted on ‘how’ to collect data using the appropriate combination of clinician- and patient-reported measures, and ‘when’ to collect data (Delphi round 4 and 5; response rates 25/25 and 25/25). During the physical face-to-face meeting (Delphi round 6; 14/25 panelists were present), consensus was reached on the previously included and outstanding inconclusive outcome domains, measurement instruments, and the timing of data collection. After the meeting, a draft ‘factsheet’ was compiled with the standard outcome set, which included: the outcome domains, the appropriate combination of PROM’s, clinician-reported outcomes measures, and the recommended timing of the data collection. The final confirmatory online vote with all panelists (Delphi round 7; response rate 25/25) was performed on this final product.

The study was performed from 2017 (preparation) through end of 2019 (data synthesis and reporting).

### Source of funding

This study was funded by a research grant from the Scoliosis Research Society.

## Results

### Part A: Outcome domains (Figs. 2, 3; Supplementary Material Table 2)

**Fig. 2:**
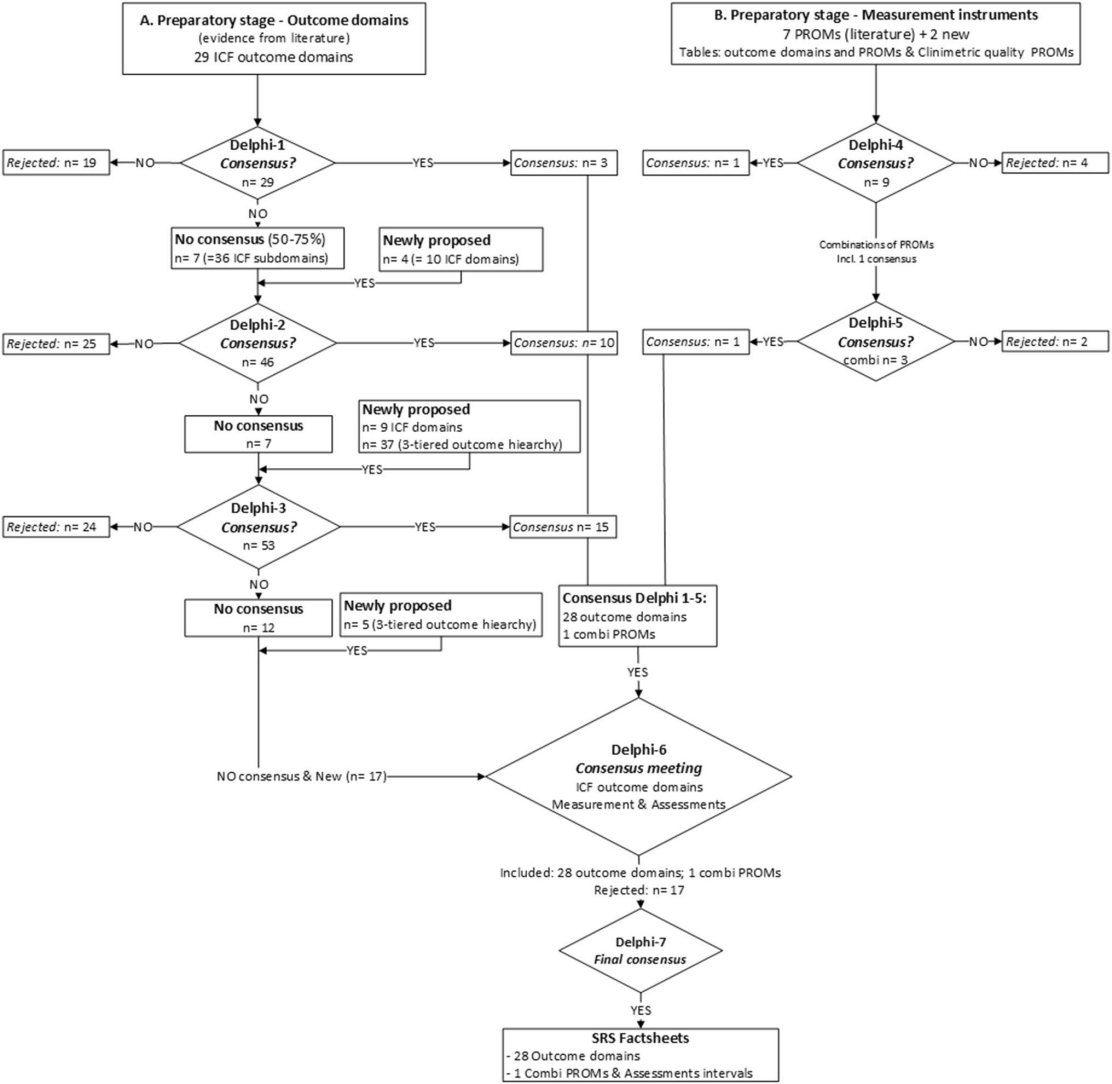
Flow of results throughout the modified Delphi procedure for outcome domains and measurement instruments. The threshold for consensus was set at ≥ 75% agreement. Items with < 50% agreement and/or repeated lack of consensus were excluded. Items with 50–75% consensus were made available again for voting in the subsequent round, and additional information from the literature was provided to make an informed decision. *Combi* refers to a combination of PROMs, *PROM* patient-reported outcome measure, *ICF* International Classification of Functioning, Disability and Health (WHO-ICF)

**Fig. 3:**
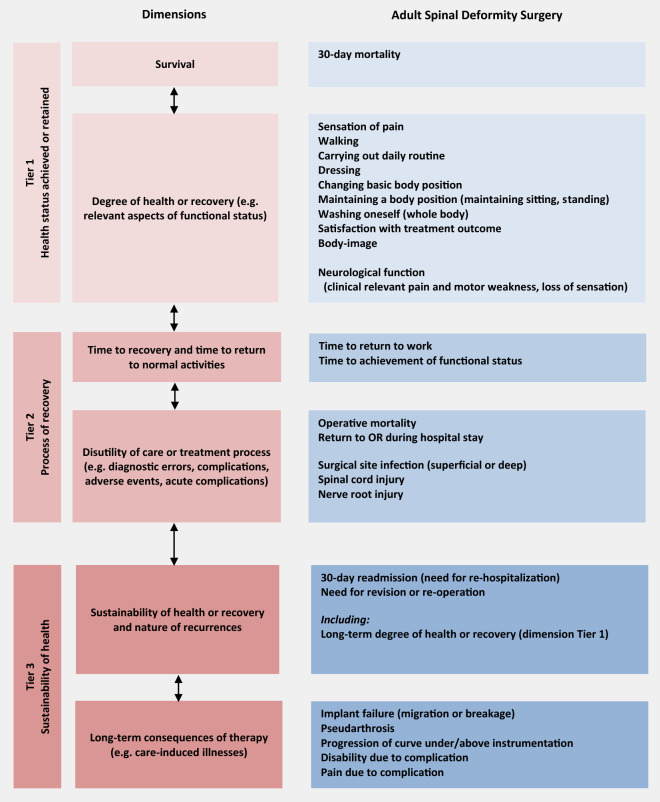
Factsheet with the outcome domains of ASD identified in this study, using the framework of the three-tiered outcome hierarchy as developed by Porter [4]

Consensus was reached to include the following domains (percentages refer to the degree of consensus):

#### Tier 1: Health status achieved or retained

*Survival* ‘30-day mortality’ (83%).

*Degree of Health or recovery* ‘sensation of pain’ (100%), ‘walking’ (92%), ‘carrying out daily routine’ (88%), ‘dressing’ (87%), ‘washing oneself (whole body)’ (75%), ‘body image’ (76%), ‘changing basic body position’ (78%) and ‘maintaining a body position’ (87%). During the Delphi process, panelists proposed the following additional domains:

- ‘Neurological function’ (NF). Several domains were identified, translated to ICF-items, and the following were voted for inclusion: NF ‘radicular pain’ (92%), NF ‘loss of sensation’ (76%), NF ‘motor weakness’ (92%),
- ‘Satisfaction with treatment outcome’ was included (96%).
- ‘Pulmonary function’: After extensive discussion and additional information from the literature, this was redefined to the ICF-item ‘exercise tolerance functions’ (b455; 54%). In Delphi round, six consensus was reached not to include this domain (86%).

#### Tier 2: Process of recovery

*Time to recovery and time to return to normal activities* ‘time to return to work’ (83%) and ‘time to achieve functional status’ (78%).

*Disutility of care or treatment process (e.g. diagnostic errors, complications, adverse events, acute complications)* During round three, the panelists reached consensus to include the most relevant adverse events in terms of severity and prevalence (Fig. 3; Tier 2–3).

#### Tier 3: Sustainability of Health

*Sustainability of health or recovery and nature of recurrences* Clinician-reported outcome domains: ‘30-day readmission’ (87%) and the ‘need for revision or re-intervention’ (97%).

*Long-term consequences of therapy (e.g. care-induced illnesses)* Longer term clinician-reported outcome domains: ‘implant failure’ (100%), ‘pseudoarthrosis’ (95%), ‘progression of curve under or above instrumentation’ (82%), ‘disability due to complication’ (82%), and ‘pain due to complication’ (77%).

### Part B. Measurement instruments and assessment intervals (Fig. 2 and Table 1)

**Table 1:** Patient-reported and clinician-reported outcomes assessments

Category	Assessments	Baseline		1 year follow up	
		Patient reported	Clinician reported	Patient reported	Clinician reported
Patient experience	EQ5D-3L	X		X	
	SRS-22r	X		X	
	ODI v2.1a	X		X	
	NPRS (0–10) Back and Leg	X		X	
	Time to return to work^a^			X	
	Time to achievement of functional status^a^			X	
Clinical status	Neurological function - Loss of sensation - Motor weakness - Radicular pain		X		X
	Operative mortality		X		
	Return to OR during hospital stay		X		
	Surgical site infection (superficial/deep)		X		
	Spinal cord injury		X		
	Nerve root injury		X		
Long-term clinical status	30-day mortality^b^				X
	30-day readmission (need for re-hospitalization)^b^				X
	Need for revision or re-intervention				X
	Implant failure (migration or breakage)				X
	Pseudoarthrosis				X
	Progression of curve under/above instrumentation				X
	Disability due to complication^a^				X
	Pain due to complication^a^				X

^a^No specific measurement method defined yet; ^b^register in primary data source (electronic medical record) after 30 days

After defining the standard set of outcome domains, the measurement instruments and assessment intervals were identified.

*Patient-reported* Many items are captured in globally available and validated composite patient-reported measurement instruments but no single PROM adequately captures all domains. During the preparatory stage, seven PROMs had been identified from the literature, and the project team added two more commonly used and reported PROMs (EQ5D-3L and AIMS2-SF; Supplementary Material Table 3) to the list distributed to the panelists during round four. After rounds four and five, consensus was reached to include the Oswestry Disability Index (ODI) v2.1a, Scoliosis Research Society (SRS)-22r, EuroQol 5-domain instrument (EQ-5D-3L), and Numeric Rating Pain Scale (NRPS [0–10]) back and leg (80%). This combination of PROMs was confirmed in the final round (96%).

*Clinician-reported* No composite instruments exist for clinician-reported outcome domains, the individual items are listed in Table 1. Notably, ‘neurological function’ is not captured with current PROMs. Following extensive face-to-face discussions during round six, the panelists reached a consensus that the items describing ‘neurological function’ (including ‘radicular pain’ [92%], ‘motor weakness’ [76%], and ‘loss of sensations’ [92%]) should be reported by the treating clinician as dichotomous (yes/no) clinical outcome domains.

### Timing of data collection

Consensus was reached to use outcome data collected at baseline (i.e. at diagnosis/index intervention) and at 1-year follow-up visit. Additional follow-up assessments at 6 months and 2 years after treatment were recommended by panelists for research purposes but not deemed mandatory for routine clinical practice.

## Discussion

Historically, older patients with symptomatic adult spinal deformity (ASD) were rarely considered candidates for surgical intervention and generally received little treatment of any kind. ASD has extremely variable presentation and some without stenosis and can result in a loss of body height, a stooped posture, and spinal stenosis. Symptoms that commonly occur are back pain, neurogenic leg pain, and fatigue due to the increased energy expenditure required to maintain an upright posture and gait. Advances in medical management, surgical technology, and changes in expectations for function and quality of life in older patients, have altered the paradigm [3]. Accordingly, surgical and nonsurgical treatments are used more. The ability to monitor and assess clinical outcomes has not kept pace.

Recent studies focusing on low back pain, such as those by the Lancet Low Back Pain Series Working Group, have emphasized the need for structured outcome data collection to facilitate risk stratification, as well as appropriate assessment of various (new) treatment strategies and resource utilization [12]. This requires the use of a standardized outcome set, but currently no such set exists for patients with ASD. This paper addresses that pressing need by providing a set of common metrics for a growing group of patients undergoing (surgical) treatment for ASD. This set should be used for continuous outcome monitoring. For clinical research, it can be further supplemented with measurements relevant to the research question.

The work was performed by a panel, employing state of the art guidelines [14], methodology [14], language [19] and framework [14, 15, 17, 19] endorsed by ICHOM [17] and OECD [18]. The current study included seven Delphi rounds administered to a panel with representation from across the globe, and achieved a very high response rate of close to 100% for the six online rounds.

The resulting outcome set covers short- and long-term health outcomes, and includes measures of disability, ability to work, and social participation [11, 12]. The full outcome set is summarized in a factsheet shown in Fig. 3. Tier one (health status achieved) covers items including 30-day mortality and degree of recovery, such as pain and activities of daily living most relevant for patients with a spinal deformity (e.g., ability to maintain an upright position). Tier two (process of recovery) reports items such as time to return to work and adverse events of the intervention (e.g. surgical site infection). Tier three (sustainability of health) reports items such as 30-day readmission, need for repeated interventions, long-term health outcomes, and late complications resulting intervention (e.g. progression of spinal deformity).

Some items required extensive review of the literature and panel discussions to identify the most appropriate ICF domains. For example, one of the major causes of disability in patients with ASD is neurogenic claudication and leg pain due to nerve root compression (radicular pain) in the deformed and degenerative lumbar spinal canal with concomitant lumbar spinal stenosis (LSS) [21]. There is disagreement on how to diagnose and test for LSS and neurogenic claudication [22]. Additionally, one of the major complications of spinal interventions is lower extremity motor loss. Previous studies have evaluated neurological function following ASD surgery using the American Spinal Injury Association (ASIA) Lower Extremity Motor Scores (LEMS) [23]. However, the ASIA-LEMS is a rigorous scoring system that has been deemed unfeasible to include in routine clinical practice. After panel discussion, it was concluded that clinician reported dichotomous (Yes/No) evaluation of ‘radicular pain’, ‘motor weakness’, ‘loss of sensation’, and patient-reported ‘walking’ ability and ‘NRS leg pain’ would adequately measure LSS, neurogenic claudication and loss of lower extremity motor function.

One important characteristic of a standard (or core) outcome set is that it should be relatively easy to obtain, using existing instruments that are available and validated in as many languages as possible. This contributes to broad acceptance and limited burden of registration, so that it can be implemented in registries, prospective cohort studies, and trials [24]. The majority of the identified domains can be captured using existing globally accepted clinician-reported outcomes (e.g. 30-day re-admission) and qualitatively adequate, globally available and accepted patient-reported outcome measures (PROMs) [20]. These include general health instruments (e.g. EQ5D-3L) and disease-specific instruments (e.g. SRS-22r).

### Outstanding issues

‘Pulmonary function’ is not covered with the proposed outcome set. ‘Pulmonary function’ appears to be an important relevant outcome for patients [25], and it has been demonstrated to deteriorate after complex ASD surgery [26, 27], but it is unclear what this domain entails and how to operationalize it. Furthermore, other as yet unknown domains may also become relevant, such as sleep deprivation, for which new (validated and reliable) measurement tools may become available. As knowledge develops (e.g. identification of new outcome domains, ranking of key outcome domains, machine learning), the standard outcome set may well need to be adapted over time. This needs to be determined in the coming years, and secured in an update cycle of this outcome set.

*Timing of data collection* The standard set we have identified requires ‘only’ 1-year follow-up, which includes shorter term outcomes such as 30-day mortality, so as to minimize the burden of data collection. Two-year follow-up is suggested for research purposes but not deemed mandatory for routine outcomes assessment. Both time-points are indeed arbitrary cut-off points, and may be considered short. It is known from all musculoskeletal pathology related to ageing that longer follow-up will change outcome, and the ASLS study [28] has demonstrated continuing changes in outcome after 3–5-year follow-up.

#### Benchmarking and risk stratification

In an ongoing related project, relevant patient characteristics (e.g. Body Mass Index) and risk factors (e.g. smoking, frailty) are being identified. These indicators are needed to allow adjustment of outcome for different patient profiles, thereby facilitating fair benchmarking across (inter)national institutions and existing outcome registries [24].

### Limitations of the current study

The panel did not have patient representation. However, one of the major challenges in creating an outcome set that addresses patient experience with global acceptance and validity is that social and cultural aspects play an important role. The aging population with a spinal deformity in Asia has quite different needs in daily life than those in North America, such as the ability to sit on the ground. Therefore, it is not yet feasible to adequately include the patient perspective on a global scale. Future validation of the current standard outcome set is recommended. Although ASD is a multimodal condition and a multidisciplinary approach would be designated, due to funding and time limitations, the panel included expert spine surgeons and spine researchers representing all continents, but no other specialties from nursing, physiatry, pain medicine, etc.

Furthermore, we cannot exclude that, as in any Delphi study, different forms of bias occurred. The risk of bias was limited, because the panel consisted of researchers and clinicians with global representation and extensive experience in the field, but not all regions of the globe were represented (e.g. Africa, Latin America) or were non-SRS members represented. On the other hand, the response rates in the Delphi rounds were close to 100%, extensive reviews of the literature were performed, and great efforts were taken strictly to follow existing methodological guidelines. Finally, the current paper has not focused on implementation in daily practice, and there are challenges in implementing questionnaires and standardized follow-up as health care becomes progressively more regulated. Issues regarding the completion rate and adoption by patients/clinicians could be very difficult in a real-world setting.

Recognizing these limitations, the authors feel the current proposed standard data set should be considered a reference for further work, for example validation in routine practice and evaluation of implementation and documentation burden.

### Future perspectives

Data collection burden is a significant hurdle to collecting standardized data, and has been the demise of many well-intentioned efforts. However, the current context seems more favorable to take the next step. Novel technology, smart-phone apps, modern EMR’s and payer/regulator requirements all favor these developments, and it is up to our spine community to now define the ‘meaningful data set’ so that we are ready to implement these technologies.

The current data set could be considered a methodical and systematic approach to patient intake for an intervention, addressing multiple domains relevant to the patient such as work, social support, and of course the medical domain. If collected, it could replace the conventional history taking in daily practice. As such, post-COVID e-health and health care away from the hospital may well form a powerful accelerator to implementation. The patient questionnaires can be applied as an online digital intake for patients, and similarly, they can be used as an online tool for the 1-year follow-up. Complemented with a follow-up X-ray, it might well allow for video consultation follow-up at 1 year instead of routine hospital visits. As such, this discrete dataset could act as a methodically collected (on-line) patient history and follow-up, it could potentially reduce current workload, and might even reduce de burden of physical patient hospital visits, while at the same time allowing for building of larger mergeable data sets that will help identify which treatment is most appropriate for which patients.

Current efforts of the American Academy of Orthopedic Surgeons (AAOS), American Association of Neurologic Surgeons (AANS) and multiple European national registries are focused on data extraction from the (electronic) medical record and from (electronically) captured patient-reported outcomes. It is to be expected that within several years most of this information will become available by automated data capture from the EMR and digital questionnaires captured on patient smartphone apps. To enhance implementation of the standard outcome set, it is recommended to involve large EMR providers. To identify why and how to facilitate data collection, the Scoliosis Research Society has recently initiated a “Platforms for Performance and Outcomes task force”.

Outcomes are regarded the end-result of care and with that they provide information on the care delivered. The proposed standard set covers the full cycle of care. When the outcome measures of the standard outcome set are implemented in routine clinical practice, they provide institutions and providers with quality outcome information of their interventions, driving Quality Improvement (QI) and value-based health care (VBHC) [29]. Value is defined as the quality and with that the (patient-reported and/or clinician-based) outcomes of an intervention, related to the costs of that intervention. These (combination of) outcomes could be used in the value equation and with that variations in care could be determined and discussed. Ultimately, the increase in uniform comparable data will allow pooling of data and this should improve the ability to identify the key drivers and essential outcomes (potentially reducing the amount of data that needs to be collected), and will aid the building of predictive analytic tools which take into account the complexity of the disease, resulting in improved (shared) decision making.

## Conclusion

This standard outcome set for patients with ASD has been developed and agreed by a panel with global representation. Using the framework of the WHO-ICF and Porter’s three-tiered outcome hierarchy the outcome domains and widely accepted measurement instruments have been identified. This framework provides a reference for further work. We recommend implementation and evaluation of the current standard set when performing clinical research. This will facilitate the future pooling of data and evaluation and optimization of the dataset. The current work has brought to light domains not routinely reported in the spinal literature (such as pulmonary function, return to work, social participation), and domains for which no adequate instruments have yet been identified (such as how to clinically quantify in routine practice lumbar spinal stenosis, neurogenic claudication, radicular pain, and loss of lower extremity motor function). The proposed set of outcome domains and corresponding measurement instruments has not yet been sufficiently validated in routine daily practice, and the documentation burden when implemented has not been evaluated. The current framework can be considered a basis for routine methodical and systematic monitoring of outcomes, potentially for all ASD patients undergoing treatment, across the world. Post-COVID e-health may accelerate the routine capture of these types of data. For this growing population of adult patients with a spinal deformity suffering a significant burden of disease, consistent reporting will increase availability of uniform data and knowledge. This will improve the ability to build decision support tools based on predictive analytics, will facilitate value driven health care, and will help identify effective interventions for the right patients. This ultimately enhances informed shared decision-making and facilitates the appropriate use of limited health care resources.

## Supplementary Information

Below is the link to the electronic supplementary material.

Supplementary file1 (PDF 649 kb)
