# Supplementary material for: The Scoliosis Research Society adult spinal deformity standard outcome set
Source: Spine Deform. 2021 Apr 6;9(5):1211–21. doi: 10.1007/s43390-021-00334-2 (PMC8363534; doi:10.1007/s43390-021-00334-2)
Supplement: Supplementary file 1 — Supplementary file1 (PDF 649 kb) [file 43390_2021_334_MOESM1_ESM.pdf]

Supplemental Figure 1. Panelists

Panelists

Twenty-five international members of the SRS agreed to participate in the Delphi study: 11 from North and Latin America, 8 from Europe and 6 from the Asia-Pacific region. The large majority of panelists were surgeons (23/25). One panelist had 5–10 years' experience (n=1, 4%), an equal number of panelists had 10–15 (n=7, 28%), 15–20 (n=7, 28%) or 20–25 (n=7, 28%) years' experience in management of or research on ASD. Finally, three panelists had 25–30 (n=3, 12%) years' experience.

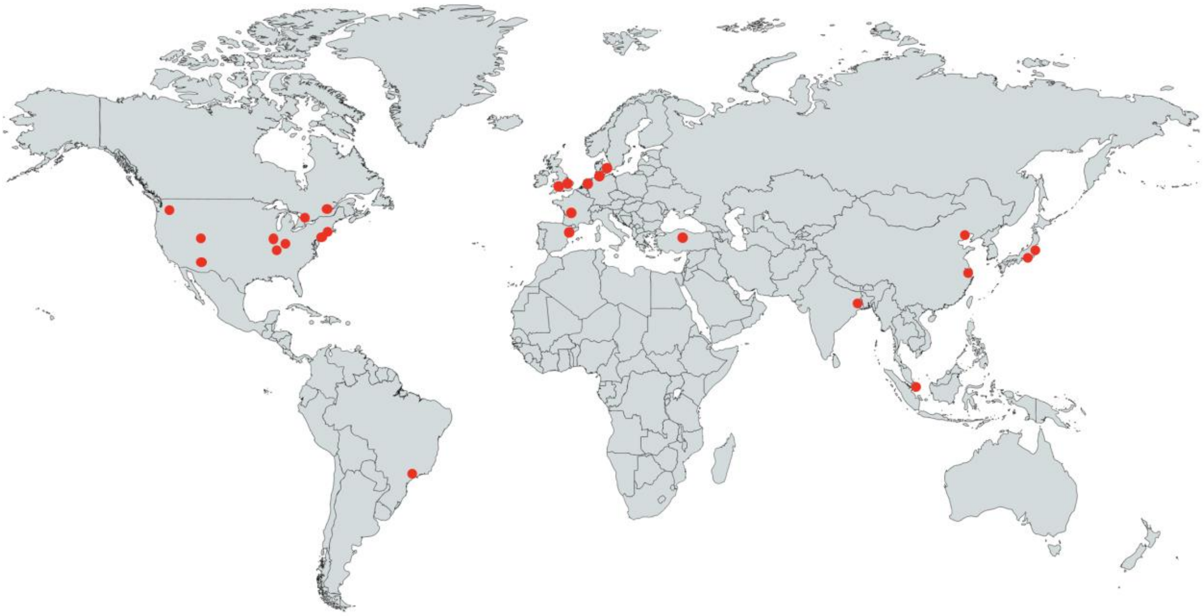

Supplemental Table 2. List of potential patient-reported and procedure-related outcome domains in the ASD surgery literature, including ICF coding and raw results of formal Delphi consensus rounds (1-3,6-7**)**

List of Potential Patient-Reported (ICF coded)[A] and Procedure-Related Outcomes [B]	% in favor for inclusion [D]
Tier 1: Health Status Achieved or Retained	
Survival [B]	
30 day mortality	83
In hospital mortality	46
Degree of Health [A]	
b280 Sensation of pain	100
d450 Walking	92
d230 Carrying out daily routine	88
d510 Washing oneself (whole body) [E]	72
- d5100 Washing body parts	33
- d5102 Drying oneself	25
- d5150 Washing whole body	75
d455 Moving around [E]	68
- d4550 Crawling	17
- d4551 Climbing	21
- d4552 Running	25
- d4554 Swimming	13
d415 Maintaining a body position [E]	87
- d4154 Maintaining a standing position	83
- d4151 Maintaining a squatting position	17
- d4152 Maintaining a kneeling position	21
- d4153 Maintaining a sitting position	79
d640 Doing housework [E]	64
- d6400 Washing and drying clothes and garments	17
- d6401 Cleaning cooking are and utensils	17
- d6402 Cleaning living area	33
- d6403 Using household appliances	25
- d6404 Storing daily necessities	17
- d6405 Disposing of garbage	21
d410 Changing basic body position [E]	78
- d4100 Lying down	46
- d4101 Squatting	17
- d4102 Kneeling	17
- d4105 Bending	33
- d4106 Shifting the body’s center of gravity	21
d540 Dressing [E]	87
- d5400 Putting on clothes	63
- d5401 Taking off clothes	54
- d5402 Putting on footwear	67
- d5403 Taking of footwear	46
- d5404 Choosing appropriate clothing	8
d920 Recreation and leisure [E]	52
- d9200 Play	21
- d9201 Sports	33
- d9202 Arts and culture	8
- d9203 Crafts	4
- d9204 Hobbies	13
- d9205 Socializing	50
d859 Work and employment, other specified and unspecified	44
b152 Emotional functions	40
b126 Temperament and personality functions	40
d430 Lifting and carrying objects	36

d850 Remunerative employment	32
d465 Moving around using equipment	32
d760 Family relationships	32
b640 Sexual function	28
d750 Informal social relationships	28
b180 Experience of self and time functions	28
b134 Sleep function	28
d710 Basic interpersonal interactions	24
e110 Products or substances for personal consumption	24
b130 Energy and drive functions	20
d445 Hand and arm use	16
d770 Intimate relationships	16
d498 Mobility, other specified	12
e165 Assets	4
e580 Health services, systems and policies	4
Neurological function (pain: radicular, claudication; covering b2803,b2803) [C]	92
Neurological function, (loss of sensation; covering b265, b2702) [C]	76
Neurological function (motor weakness; covering b5253, b6202, b7300) [C]	92
Satisfaction with treatment outcome (environmental domain, not covered in ICF)[C]	96
Satisfaction with symptom state (not covered in ICF) [C]	60
Body image (b1801; representation and awareness of one's body) [C]	76
Pulmonary function (wheezing; covering b460) [C]	0
Pulmonary function (coughing; covering b450) [C]	0
Pulmonary function (phlegm; not covered in ICF) [C]	0
Pulmonary function (shortness of breath during physical activities; covering b455 excercise tolerance functions) [C]	54
**Tier 2: Process of Recovery**	
**Time to Recovery and Time to Return to Normal Activities** [B]	
- Time to surgical treatment	39
- Time to return to social activities	57
- Time to return to physical activities	65
- Time to return to work	83
- Time to achievement of functional status	78
- Time to achievement of cosmetic status	13
**Disutility of Care or Treatment Progress** [B]	
- Length of hospital stay	61
- Return to OR during hospital stay	87
- Operative mortality	97
- Pneumonia	39
- Pulmonary embolus	61
- Urinary tract infection	35
- Urinary retention	30
- Ileus	30
- Hemorrhage / Hematoma	70
- Wound infection	91
- Nerve root injury	83
- Dural tear	43
- Implant malposition	70
- Spinal cord injury	91
- Deep vein thrombosis	61
- Myocardial infarction	57
- Blindness	74
- Sepsis	48
- Delirium	26
**Tier 3: Sustainability of Health**	
**Sustainability of Health or Recovery and Nature of Recurrences** [B]	

- 30 day readmission (need for re-hospitalization)	87
- Need for revision or re-operation	97
Long-term Consequences of Therapy [B]	
- Disability due to complication	82
- Pain due to complications	77
- Continuous per-oral analgesic use	32
- Implant failure (migration or breakage)	100
- Pseud arthrosis	95
- Vertebral compression fracture	59
- Progression of curve under or above instrumentation	82
- Susceptibility to infection	14

[A] List of patient-reported outcomes (ICF coded) in ASD surgery were derived from the following literature review:

- Faraj SSA, van Hooff ML, Holewijn RM, et al (2017) Measuring outcomes in adult spinal deformity surgery: a systematic review to identify current strengths, weaknesses and gaps in patient-reported outcome measures. Eur Spine J 26: . doi: 10.1007/s00586-017-5125-4

[B]List of procedure-related outcomes in ASD surgery was derived from the following studies:

- Baron EM, Albert TJ. Medical complications of surgical treatment of adult spinal deformity and how to avoid them. Spine (Phila Pa 1976) 2006;31:S106-18
- Di Capua J, Somani S, Kim JS, et al. Hospital-Acquired Conditions in Adult Spinal Deformity Surgery: Predictors for Hospital-Acquired Conditions and Other 30-Day Postoperative Outcomes. Spine (Phila Pa 1976) 2017;42:595–602
- Manoharan SR, Baker DK, Pasara SM, et al. Thirty-day readmissions following adult spinal deformity surgery: an analysis of the National Surgical Quality Improvement Program (NSQIP) database. Spine J 2016;16:862–6
- Soroceanu A, Diebo BG, Burton D, et al. Radiographical and Implant-Related Complications in Adult Spinal Deformity Surgery. *Spine (Phila Pa 1976)* 2015;40:1414–21
- Lapp MA, Bridwell KH, Lenke LG, et al. Long-term complications in adult spinal deformity patients having combined surgery: A comparison of primary of revision patients. Spine (Phila Pa 1976) 2001;26:973–83
- Clement RC, Welander A, Stowell C, et al. A proposed set of metrics for standardized outcome reporting in the management of low back pain. Acta Orthop. 2015;86(4):1-11. doi:10.3109/17453674.2015.1036696.

[C] Outcomes proposed by panelists and project team following round 1 and 2.

[D] Outcomes with 50–75% consensus were made available again for voting in the subsequent round.

- Percentages indicate final voting results.

[E] ICF 2nd level outcome with 50-75% in favor, were transferred to ICF 3rd level outcome in the consecutive round to achieve more granularity.

Supplemental Table 3 Patient-reported outcome measures; domain coverage

	PROMs identified in the literature							PROMs proposed by research team / panelists	
Outcomes in Standard Set	ODI V2.1a	SRS-22r	SF-12	SF-36	SRS-24	SRS-30	NRPS	AIMS2-SF	EQ-5D
Carrying out daily routine	total score	5, 12	2,5	4, 15, 16	4,9	5,12		25	3
Sensation of pain	1	1, 2, 8, 17	8	21, 22	1,2,6, 10,18	1,2,8,27	1	13	4
Changing basic body position	5,6			8	4			2, 12	
Maintaining body position	5, 6							2	
Walking	4	5		9, 10, 11	4	5		4	1
Washing oneself	2			12					2
Dressing	2			12				7, 11	2
Neurological function[A]									
Body Image		6, 10, 19							
Satisfaction with treatment outcome		4, 21, 22			3, 22, 24	21,22			

ODI indicates Oswestry Disability Index version 2.1a, SRS-22r: Scoliosis Research Society version 22r, SF: Short Form, NRPS: Numeric Rating Pain Scale, AIMS: Arthritis Impact Measurement Scale, EQ-5D: EuroQol-5 Domains

Reported numbers indicate questionnaire items. In green are outcome domains covered by PROMs.

[A] Outstanding issue discussed during face-to-face meeting. The panelists reached consensus (100%) that items of neurological function (including clinically relevant neurological pain, motor weakness, and loss of sensations) should be collected by the treating physician.
